# Supplementary material for: Controllable Preparation of SERS-Active Ag-FeS Substrates by a Cosputtering Technique
Source: Molecules. 2019 Feb 2;24(3):551. doi: 10.3390/molecules24030551 (PMC6384828; doi:10.3390/molecules24030551)
Supplement: Supplementary file 1 [file molecules-24-00551-s001.pdf]

Supplementary Materials

Controllable Preparation of SERS-active Ag-FeS Substrate by Cosputtering Technique

Ning Ma[1], Xin-Yuan Zhang[2], Wenyue Fan[1], Bingbing Han[1], Sila Jin[3], Yeonju Park[3], Lei Chen[1,*], Yongjun Zhang[2,*], Yang Liu[2], Jinghai Yang[2] and Young Mee Jung[3,*]

[1] Key Laboratory of Preparation and Applications of Environmental Friendly Materials, Ministry of Education, College of Chemistry, Jilin Normal University, Changchun 130103, P.R. China; elva_maning@163.com (N.M.); 15504341371@163.com (W.F.); bingbinghan0106@163.com (B.H.); chenlei@jlnu.edu.cn (L.C.)

[2] Key Laboratory of Functional Materials Physics and Chemistry, Ministry of Education, College of Physics, Jilin Normal University, Changchun 130103, P.R. China; zxyciomp@126.com (X.-Y.Z.); liuyang@jlnu.edu.cn (Y.L.); jhyang1@jlnu.edu.cn (J.Y.); yjzhang@jlnu.edu.cn (Y.Z.)

[3] Department of Chemistry, Institute for Molecular Science and Fusion Technology, Kangwon National University, Chunchon 24341, Korea; jsira@kangwon.ac.kr (S.J.); yeonju4453@kangwon.ac.kr (Y.P.); ymjung@kangwon.ac.kr (Y.M.J.)

* Correspondence: chenlei@jlnu.edu.cn (L.C.); yjzhang@jlnu.edu.cn (Y.Z.); ymjung@kangwon.ac.kr (Y.M.J.); Tel: +82-33-250-8495

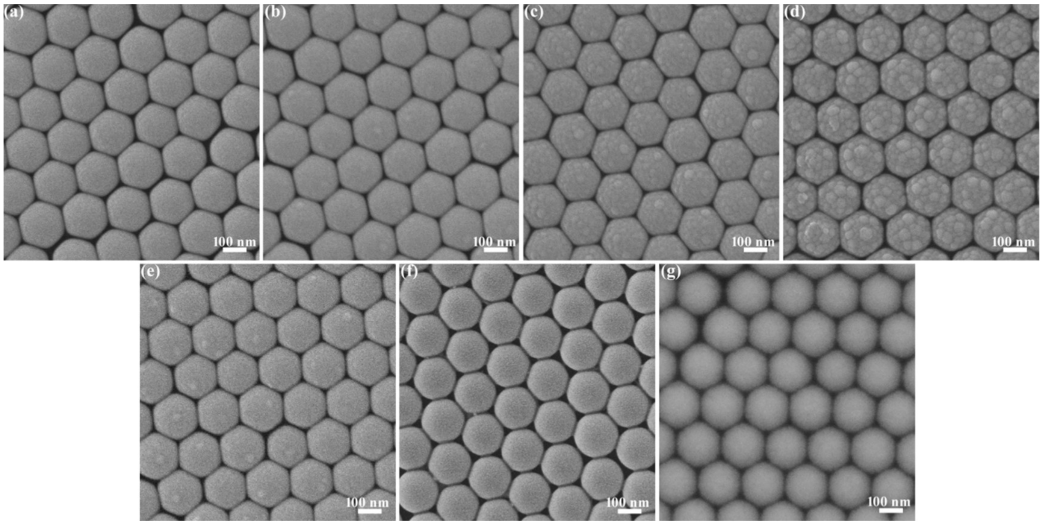

Figure S1. SEM images of the ordered Ag-FeS arrays, which were prepared by cosputtering Ag with a constant sputtering power (5 W) and FeS with varied sputtering powers for 300 s on the PSCP templates. The sputtering powers of FeS were (a) 50 W, (b) 60 W, (c) 70 W, (d) 80 W, and (e) 90 W; sputtered pure FeS (f); and pure Ag (g) for 300 s.
